# Effectiveness of the Pediatric Nursing Excellence Model on Nurses’ Knowledge and Practice in Pediatric Orthopedic Surgery Care: A Randomized Controlled Trial

**DOI:** 10.3390/children11121457

**Published:** 2024-11-29

**Authors:** Osama Mohamed Elsayed Ramadan, Alaa Hussain Hafiz, Nadia Bassuoni Elsharkawy, Nouran Essam Katooa, Areej Abunar, Enas Mahrous Abdelaziz, Samia Ibrahim Mabrouk Baraka, Mostafa Shaban, Nagwa Ibrahim Mabrouk Baraka

**Affiliations:** 1 Department of Maternal and Child Health Nursing, College of Nursing, Jouf University, Sakaka 72388, Al Jouf, Saudi Arabia; nelsharkawy@ju.edu.sa; 2 Department of Maternity and Child Health, Faculty of Nursing, King Abdulaziz University, Jeddah 22254, Saudi Arabia; ahhafidh@kau.edu.sa (A.H.H.); nkuttouaha@kau.edu.sa (N.E.K.); aabunaar@kau.edu.sa (A.A.); 3 Department of Psychiatric Mental Health Nursing, College of Nursing, Jouf University, Sakaka 72388, Al Jouf, Saudi Arabia; emabdelhamid@ju.edu.sa; 4 Department of Community Health Nursing, Faculty of Nursing, Tanta University, Tanta 31527, Egypt; samya_baraka@nursing.tanta.edu.eg; 5 Department of Community Nursing, College of Nursing, Jouf University, Sakaka 72388, Al Jouf, Saudi Arabia; mskandil@ju.edu.sa; 6 Department of Pediatric Nursing, Faculty of Nursing, Tanta University, Tanta 31527, Egypt; nagwa_baraka@nursing.tanta.edu.eg

**Keywords:** pediatric nursing, orthopedics surgery, nursing education, professional excellence, randomized controlled trial

## Abstract

Background: Pediatric orthopedic nursing requires specialized competencies to optimize patient outcomes, particularly in the complex realm of pediatric surgery. This study explores the effectiveness of the Pediatric Nursing Excellence (PNE) Model in enhancing nurses’ knowledge and clinical practice in providing perioperative care for pediatric orthopedic patients in tertiary care inpatient settings. Methods: A double-blind, randomized controlled trial was conducted from February to July 2024, involving 100 nurses from two tertiary care hospitals in Tanta, Egypt. Participants were randomly assigned to receive PNE Model training (*n* = 50) or routine care (*n* = 50). Nurses’ knowledge, practical skills, and adherence to PNE principles were assessed at baseline, immediately post-intervention, and after one, three, and six months using validated tools. Results: The intervention group showed significantly greater improvements in knowledge (20.62 ± 6.7 vs. 8.16 ± 5.5, *p* < 0.001) and practice scores (62.28 ± 4.1 vs. 40.06 ± 14.7, *p* < 0.001) post-intervention, sustained over six months. Path analysis revealed that the PNE Model enhanced nursing practice directly (β = 0.25, *p* < 0.001) through improvements in engagement and adherence to excellence principles and indirectly (β = 0.53, *p* < 0.001) by significantly enhancing nurses’ knowledge (β = 0.70, *p* < 0.001), which in turn positively influenced their clinical practice (β = 0.75, *p* < 0.001). Post-intervention, 82% of nurses in the intervention group achieved high levels of professional excellence, compared to 8% at baseline (*p* < 0.001). Conclusions: The PNE Model demonstrates robust effectiveness in enhancing nurses’ knowledge, clinical practice, and professional excellence in pediatric orthopedic surgery care, with sustained long-term benefits. This evidence supports implementing specialized nursing education models to improve pediatric care quality in orthopedic settings.

## 1. Introduction

Pediatric orthopedic surgery addresses musculoskeletal disorders such as developmental dysplasia of the hip, clubfoot, scoliosis, and fractures, which are significant contributors to pediatric hospital admissions [1,2]. These conditions demand specialized care due to children’s unique physiological and psychological needs, requiring advanced skills from healthcare providers, particularly nurses [3,4]. Reynolds et al. (2023) emphasized that nurses play a pivotal role in optimizing perioperative and postoperative care in pediatric orthopedic surgery, ultimately influencing patient outcomes [5].

Pediatric orthopedic surgery presents unique challenges that extend beyond surgical precision, requiring a multidisciplinary approach to manage both physical and emotional aspects of care [6,7,8]. These challenges include the complexity of perioperative care for musculoskeletal conditions, the need for advanced surgical skills, and the high reliance on evidence-based protocols to prevent complications such as infections, delayed healing, and reduced mobility [3,9,10]. Additionally, children undergoing surgery often experience heightened levels of anxiety and fear, which, if not managed effectively, can hinder recovery [11]. Tailored approaches that integrate age-appropriate education, effective communication, and emotional support into care protocols have been shown to reduce postoperative complications by up to 30%, according to a meta-analysis by Sun et al. (2023) [12]. However, the consistent implementation of these strategies relies heavily on the expertise of the nursing staff, who play a critical role in addressing the physical, emotional, and developmental needs of children while actively involving families in rehabilitation and recovery [13]. The Pediatric Nursing Excellence (PNE) Model was developed to address these challenges by equipping nurses with specialized competencies and structured care strategies to optimize outcomes in this high-demand setting [14]. In contrast to other pediatric specialties, orthopedic surgery requires nursing care that integrates advanced technical skills with a strong emphasis on long-term rehabilitation and mobility recovery [15].

Nurses play a central role in the multidisciplinary care of pediatric orthopedic patients [16,17]. Their responsibilities encompass the entire surgical care continuum, from preoperative preparation and intraoperative support to postoperative monitoring and discharge planning [18,19]. Furthermore, their direct involvement in pain management, wound care, and patient education positions them as key contributors to patient outcomes [20,21]. Choi et al. (2022) demonstrated that hospitals with a higher proportion of specialized pediatric orthopedic nurses had 25% fewer postoperative complications and 20% shorter hospital stays [22]. This underscores the critical need for specialized nursing skills in this field [23]. Despite their vital role, nurses in pediatric orthopedic units face numerous challenges that limit the consistent delivery of high-quality care [24]. A key issue is the lack of access to specialized training programs tailored to pediatric orthopedic nursing [25,26]. Additionally, variations in clinical practice standards, high patient-to-nurse ratios [27], the number of weekly shifts and the total working hours of nurses further complicate the provision of consistent, evidence-based care [28,29,30]. Prolonged working hours and frequent shifts contribute to fatigue and reduced job satisfaction, which may compromise clinical performance and patient outcomes [31]. In a survey conducted by the International Pediatric Orthopedic Nursing Association (2023), only 45% of nurses reported having received specialized training in pediatric orthopedics, highlighting a significant gap in professional development opportunities [14]. This gap is not merely an issue of professional growth; it directly affects patient care outcomes and family satisfaction [32,33].

In response to these, the PNE Model was developed and preliminarily validated in prior studies to enhance nursing practice in pediatric orthopedic care [14]. Validation efforts focused on its theoretical alignment with Benner’s Novice to Expert theory, the Quality-Caring Model, and its applicability in clinical training programs [14,34,35]. The PNE Model is grounded in two foundational theories, Benner’s Novice to Expert theory and the Quality-Caring Model, providing a comprehensive framework for developing nursing expertise and fostering relational care [36,37,38]. Benner’s Novice to Expert theory emphasizes the progression of nursing expertise through experiential learning, which is particularly crucial in specialized fields like pediatric orthopedic surgery, where nurses must navigate complex clinical scenarios [39,40]. This theory supports the PNE Model’s focus on skill acquisition through structured, hands-on training and continuous professional development [41]. The Quality-Caring Model complements this by emphasizing the importance of interpersonal relationships and caring practices in achieving optimal outcomes [42], which is critical in pediatric care, where emotional and family support significantly influence recovery [43].

The PNE Model uniquely addresses pediatric orthopedic care through four components: evidence-based practices, mentorship, simulation-based learning, and family-centered care [14,44]. Evidence-based practices ensure standardized and up-to-date approaches to perioperative care, improving patient safety and clinical outcomes [45]. Simulation-based learning provides opportunities for nurses to practice complex surgical procedures in controlled environments, bridging the gap between theoretical knowledge and practical application [45,46]. Mentorship accelerates skill acquisition through guided experience, fostering professional growth and teamwork [44]. The family-centered approach emphasizes active family participation in perioperative care, fostering better outcomes and higher satisfaction levels [47,48]. The PNE Model thus bridges theoretical knowledge and practical application while addressing pediatric patients and their families’ physical, emotional, and psychological needs. Integrating evidence-based care, mentorship, simulation, and family involvement provides a targeted strategy for improving nursing competencies and advancing care quality in pediatric orthopedic surgery [44].

**Figure 1:**
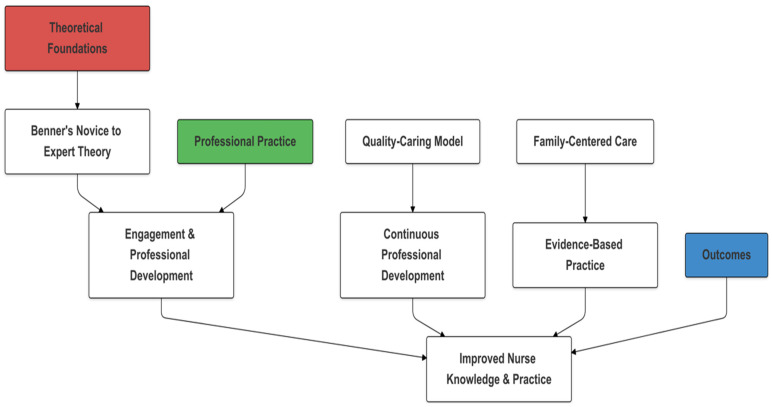
Conceptual framework of the Pediatric Nursing Excellence (PNE) Model in pediatric orthopedic care.

Figure 1 illustrates the conceptual framework of the PNE Model, which highlights the progression from theoretical foundations to practical implementation and expected outcomes. This framework demonstrates how the integration of the novice-to-expert theory and the Quality-Caring Model supports the development of specialized pediatric care competencies, leading to improved nurse knowledge, practice, and patient outcomes. The PNE Model’s emphasis on professional development and family-centered care is particularly relevant in pediatric orthopedic settings, where patient recovery is influenced by the nurse’s technical proficiency and ability to communicate effectively with children and their families. This holistic approach ensures that nurses are equipped to handle the clinical aspects of pediatric orthopedic care and their patients’ emotional and psychological needs.

Despite the robust theoretical grounding of the PNE Model, empirical evaluations of its effectiveness in pediatric orthopedic nursing are limited [44,49]. Mlambo et al. (2023) identified only a few studies that explored the impact of structured nursing models on knowledge acquisition and practical skill development in pediatric orthopedics, none of which focused on the comprehensive application of a model like PNE [37]. This gap in the literature underscores the need for rigorous studies to determine how nursing excellence models like PNE influence both nursing competencies and patient outcomes in specialized pediatric care settings [44,50].

## 2. Materials and Methods

### 2.1. Research Questions

This study aimed to address the following research questions:

#### 2.1.1. Primary Research Question

1. Does the implementation of the Pediatric Nursing Excellence (PNE) Model significantly improve nurses’ knowledge and practice in pediatric orthopedic surgery care compared to conventional training methods?

#### 2.1.2. Secondary Research Questions

1. Does the PNE Model lead to sustained improvements in nurses’ knowledge over time after the intervention?
2. To what extent does the PNE Model enhance nursing practice and clinical skills in pediatric orthopedic surgery care?
3. Is there a positive correlation between the increases in nurses’ knowledge and the improvements in their practical skills following the PNE Model intervention?

### 2.2. Research Hypothesis

We hypothesized that nurses who received training based on the PNE Model would demonstrate greater improvements in both knowledge and practice than those who did not. Specifically, we predict the following:

**H1.**  *Nurses in the intervention group will show significantly higher scores on knowledge assessments post-intervention compared to the control group*.

**H2.**  *The intervention group will demonstrate superior performance in clinical practice, as measured by standardized observational checklists*.

**H3.**  *There will be a positive correlation between increases in knowledge and enhancements in clinical practice skills among nurses trained in the PNE Model*.

### 2.3. Design

This study used a double-blind, randomized controlled trial (RCT) to evaluate the effectiveness of the Pediatric Nursing Excellence (PNE) Model on nurses’ knowledge and practice in caring for children undergoing orthopedic surgery. Data collection spanned from 1 February 2024 to 30 July 2024, enabling the assessment of both immediate and sustained effects of the intervention. An RCT design was chosen as it was considered the gold standard for determining causal relationships between interventions and outcomes in healthcare research [51]. The random allocation of participants to intervention or control groups, performed using a computer-generated sequence, minimized selection bias and ensured that any observed differences could be attributed to the intervention rather than pre-existing disparities. Although stratification was not used, randomization aimed to balance key demographic and clinical characteristics between groups. Randomization also controlled for both known and unknown confounding variables by distributing them evenly across groups, enhancing the internal validity of the study. Additionally, the double-blind approach reduced performance and detection bias, as both participants and outcome assessors were unaware of group assignments. The inclusion of a control group receiving routine care provided a basis for comparison, allowing for an accurate assessment of the PNE Model’s effectiveness relative to existing practices. Randomization checks confirmed the balance between baseline characteristics of both groups. This design established a strong foundation for evaluating the PNE Model’s effectiveness and its potential to enhance the quality of care for pediatric patients undergoing orthopedic surgery in tertiary care inpatient settings, contributing valuable insights to specialized nursing education.

### 2.4. Settings

The study was conducted at two tertiary care institutions in Tanta, Egypt, both specializing in pediatric orthopedic surgery and providing comprehensive perioperative care for children with musculoskeletal conditions. The first setting was the Orthopedic Department of the New Surgical Hospital, Tanta University, which has a total capacity of 250 beds, including 30 beds specifically dedicated to orthopedic services. This hospital is recognized for its advanced surgical capabilities, high patient volumes, and a strong commitment to professional development, making it an ideal setting for implementing specialized nursing interventions. The second setting was the Pediatric Surgical Orthopedic Department of Tanta Educational Hospital, which operates within a facility with a total capacity of 200 beds, of which 25 are allocated exclusively for pediatric orthopedic care. Known for its focus on pediatric surgical excellence, this hospital manages a diverse range of complex orthopedic cases. These distinct yet complementary institutions were chosen to strengthen the study’s external validity by providing a diverse and representative sample of pediatric patients and nursing staff. Their specialization in pediatric orthopedic surgery, advanced perioperative care capabilities, and emphasis on professional development among healthcare providers made them well-suited for assessing the effectiveness of the Pediatric Nursing Excellence (PNE) Model in real-world clinical environments. By utilizing these settings, the study aimed to gain valuable insights into the broader applicability of the PNE Model in pediatric orthopedic nursing across varied institutional contexts.

### 2.5. Participant Sample Size Determination

The sample size was calculated using G*Power software (version 3.1.9.7), a widely recognized tool for statistical power analysis [52]. A *t*-test for independent samples was employed, assuming a medium effect size (Cohen’s d = 0.5), a power of 0.80, and a significance level of 0.05. These parameters were selected based on prior studies assessing educational interventions in nursing practice, which reported similar effect sizes [53,54,55,56]. An alpha level of 0.05 ensures a 5% risk of a Type I error, while a power of 0.80 minimizes the likelihood of a Type II error, providing an 80% probability of detecting a statistically significant effect if one exists [57]. The analysis determined that a minimum of 80 nurses was required, with 40 participants in the intervention group (trained in the Pediatric Nursing Excellence [PNE] Model) and 40 in the control group (receiving routine care). Equal allocation was chosen to maximize statistical power and simplify analysis. Randomization was conducted using a computer-generated random sequence, employing simple randomization to ensure unbiased group allocation. Stratification by site was deemed unnecessary due to the comparable characteristics of the two hospitals involved. An additional 20% was added to the calculated sample size to account for potential attrition or non-compliance. Therefore, the final sample size was adjusted to 100 nurses, with 50 nurses allocated to the intervention group and 50 to the control group. Randomization was conducted using a computer-generated random sequence, employing simple randomization to ensure unbiased group allocation. This approach maintained equal group sizes, enhancing the study’s internal validity.

### 2.6. Eligibility Criteria

Inclusion Criteria:

- Registered nurses currently employed in the pediatric orthopedic departments of the participating hospitals.
- Minimum of one year of clinical experience in pediatric nursing.
- Full-time employment status (minimum 36 h per week).
- Willingness to participate in the study for its full duration, including follow-up assessments.

Exclusion Criteria:

- Nurses in managerial or exclusively administrative roles with no direct patient care responsibilities.
- Nurses on extended leave (e.g., maternity, sick leave) during the study period.
- Participation in any other formal pediatric orthopedic nursing education program concurrent with the study.

**Figure 2:**
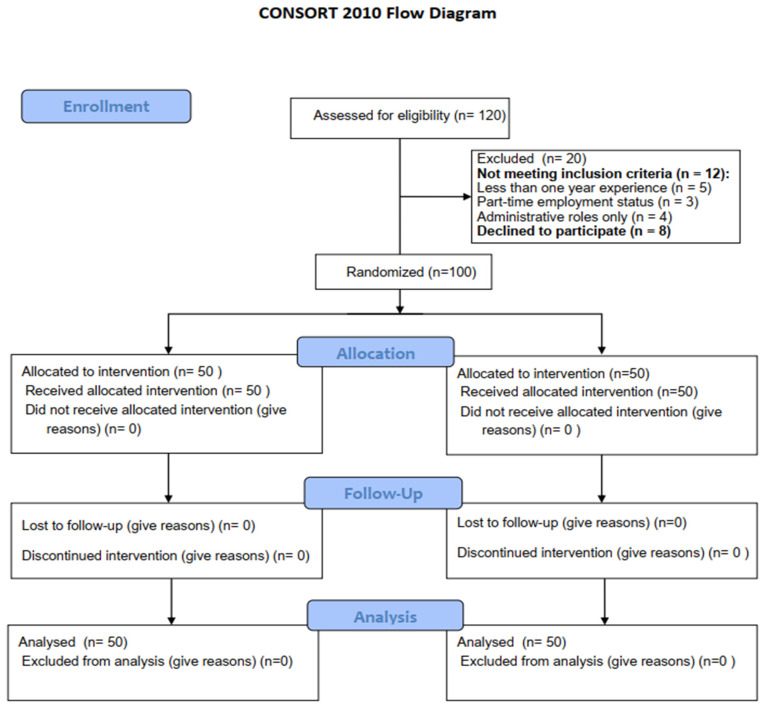
Study flow chart.

Based on the specified inclusion and exclusion criteria, a total of 120 nurses were assessed for eligibility to participate in the study. As depicted in Figure 2, 20 nurses were excluded: 12 did not meet the inclusion criteria (5 had <1 year experience, 3 were part-time, and 4 were in administrative roles), and 8 declined to participate. The remaining 100 nurses were randomly assigned equally to either the intervention group (*n* = 50) or the control group (*n* = 50). All participants completed the study without any losses to follow-up or withdrawals.

### 2.7. Instruments

Four primary instruments were used to collect data in this study: (1) the Participant Characteristics Questionnaire, (2) the Nurses’ Knowledge Assessment Questionnaire, (3) the Observational Checklist for Nursing Practice, and (4) the Pediatric Nursing Excellence (PNE) Model Questionnaire. Each tool was meticulously developed and validated to ensure accurate measurement of the key constructs: nurses’ knowledge, practice skills, and adherence to the PNE Model principles in pediatric orthopedic surgery care. A pilot study involving 10% of the nurses was conducted to assess the tools’ clarity, applicability, and feasibility. Based on the pilot study results, necessary modifications were made, and the pilot data were excluded from the final analysis.

1. Participant Characteristics Questionnaire:

This tool gathered contextual data about both the nurses and the pediatric patients under their care to provide insights into the background of study participants. It was developed based on an extensive review of current literature and existing validated instruments in the field of pediatric nursing and orthopedic care [58]. The questionnaire comprised demographic data of nurses. This section collected information on the nurses’ personal and professional backgrounds, such as age, gender, educational qualifications, years of nursing experience, and prior training related to pediatric orthopedic care. These data provided valuable insights into the characteristics of the participants.

2. Nurses’ Knowledge Assessment Questionnaire:

The Nurses’ Knowledge Assessment Questionnaire consisted of 24 multiple-choice questions designed to evaluate essential topics in pediatric orthopedic nursing, including definitions of common conditions, causes, types of surgeries, diagnostic procedures, complications, preoperative and postoperative care principles, strategies for preventing complications, caregiver education, and discharge planning. Content validity was ensured through review by a panel of five experts in pediatric nursing and orthopedic care, who evaluated item relevance, clarity, and comprehensiveness, resulting in a Content Validity Index (CVI) of 0.95, indicating excellent agreement [59]. Reliability was assessed using Cronbach’s alpha, which yielded a coefficient of 0.85, reflecting good internal consistency and ensuring the tool reliably measured the intended knowledge domain [60]. Scoring was based on a 0–48 point scale, with 2 points for correct answers, 1 point for partially correct answers, and 0 points for incorrect or “don’t know” responses. Knowledge levels were categorized as low (<60%, <29 points), moderate (60–79%, 29–38 points), and high (≥80%, 39–48 points).

3. Observational Checklist for Nursing Practice:

The Observational Checklist for Nursing Practice assessed nurses’ practical skills and competencies in caring for pediatric orthopedic surgery patients [61,62,63]. Adapted from established tools and guidelines in pediatric orthopedic nursing, the checklist comprised 73 items across four domains: Preoperative Care (13 items), addressing patient assessment, surgery preparation, informed consent, and preoperative education; Transfer Care (5 items), covering safe patient transfer to the operating room, continuity of care, and patient safety; Postoperative Care (46 items), encompassing vital sign monitoring, pain management, wound care, neurovascular assessments, complication prevention, early mobilization, and documentation; and Health Education and Discharge Planning (9 items), focused on educating patients and families about postoperative care, medication, activity restrictions, complications, and follow-up. Content validity, evaluated by the same expert panel as the knowledge questionnaire, achieved a CVI of 0.95 [59]. Reliability was demonstrated with a Cronbach’s alpha of 0.93, indicating excellent internal consistency, and inter-rater reliability yielded a Cohen’s kappa coefficient of 0.88, reflecting strong agreement [14]. Scoring assigned 1 point for correctly performed items and 0 for incorrect or omitted actions, with total scores ranging from 0 to 73. Practice levels were classified as satisfactory (≥58 points, ≥80%) or unsatisfactory (<58 points, <80%).

4. Pediatric Nursing Excellence (PNE) Model Questionnaire:

The PNE Model Questionnaire was designed to evaluate nurses’ adoption of principles from the Pediatric Nursing Excellence (PNE) Model [14]. Adapted from the original framework and related literature, the instrument was translated into Arabic and back-translated into English to ensure linguistic and conceptual equivalence [64]. It included 28 statements across five domains: Engagement (assessing teamwork, professionalism, and commitment to professional development), Values (evaluating advocacy, ethical adherence, and quality-of-life improvements), Principles (measuring equity, holistic care, and family-centered practices), Care Delivery (focusing on coordination, care planning, and health promotion), and Continuous Improvement (assessing evidence-based practices, patient outcomes, and quality standards). Responses were rated on a three-point Likert scale: Agree (2 points), Neutral (1 point), and Disagree (0 points). Content validity, evaluated by experts, achieved a CVI of 0.95, indicating strong relevance and clarity. Reliability testing via Cronbach’s alpha yielded a coefficient of 0.82, reflecting good internal consistency [65]. The scoring system ranged from 0 to 56 points, with nurses’ excellence levels classified as low (<28 points, <50%), moderate (28–41 points, 50–74%), or high (≥42 points, ≥75%).

### 2.8. Ethics Approval

Ethical approval for this study was obtained from the Ethical Committee of the Faculty of Nursing, Tanta University, on 17 December 2023 (Approval No. 358-12-2023), prior to the commencement of the research. The study was registered with ClinicalTrials.gov (Identifier: NCT06624007). Additionally, official permission was secured from the manager of the Pediatric Hospital at Tanta University Hospital. This ensured adherence to institutional and ethical guidelines. The study was designed with careful consideration of ethical and legal principles to protect the rights and welfare of participants. It posed no risk of harm or discomfort to the nurses involved, as the nature of the research was centered on educational interventions and observational data collection.

Confidentiality and privacy were strictly maintained throughout the study, with participant information anonymized and securely stored to prevent unauthorized access. Written informed consent was obtained from all participants, ensuring they were fully aware of the study’s aims, procedures, and potential benefits. Participants were also informed of their right to withdraw from the study at any time without any adverse consequences to their professional standing or employment. These ethical safeguards ensured that the study was conducted in accordance with the highest standards of research integrity and participant protection.

### 2.9. Study Phases and Procedures

The study was conducted in four methodical phases: Assessment, Planning, Implementation, and Evaluation, based on a rigorous randomized controlled trial (RCT) framework. This structure was meticulously designed to evaluate the impact of the Pediatric Nursing Excellence (PNE) Model on nurses’ knowledge and clinical practice in pediatric orthopedic surgery care. The study took place across two tertiary hospitals in Tanta, Egypt, from February to July 2024.

#### 2.9.1. Randomization and Group Allocation

A total of 100 nurses were recruited and randomly assigned to two groups using a computer-generated random sequence to ensure demographic and professional balance. The intervention group (*n* = 50) received specialized training based on the Pediatric Nursing Excellence (PNE) Model, while the control group (*n* = 50) continued routine pediatric orthopedic nursing care. Each nurse cared for an average of two pediatric patients throughout the study, maintaining a standardized nurse-patient workload to minimize confounding variables and ensure that changes in knowledge and practice could be attributed solely to the intervention. Randomization was conducted using a computer-generated sequence, and demographic balance assessments confirmed comparability between the groups. Both nurses and outcome assessors were blinded to group assignments to prevent performance and detection biases, enhancing the study’s internal validity. Blinding protocols included concealing group allocation details from participants and assessors, with only study coordinators having access to this information. This rigorous approach ensured unbiased delivery and evaluation of the intervention.

Multiple blinding measures were implemented to maintain the integrity of the double-blind, randomized controlled trial. For participant blinding, the training materials for both groups were designed with identical packaging and labeled as Professional Development Program A and Professional Development Program B. These materials were prepared and distributed by an independent research coordinator who was not involved in delivering the intervention or assessing outcomes. The intervention and control group training sessions were conducted in separate facilities at distinct times to avoid cross-contamination. All participants were informed they were part of a professional development initiative but were not made aware of their specific group assignment. Although we cannot entirely rule out the possibility of informal discussions among nurses revealing group assignments, participants were explicitly instructed to avoid sharing details about their training. Additionally, the separate timing and location of sessions, combined with blinded outcome assessments and statistical analyses, were designed to mitigate this risk and ensure the validity of study findings. For investigator blinding, trainers were divided into two independent teams, each assigned exclusively to deliver either the Pediatric Nursing Excellence (PNE) Model training or the control training. Each team was thoroughly trained in their respective program’s content but was not provided access to the content of the alternative program. Trainers were instructed not to discuss program details with colleagues outside their designated team to minimize the risk of unblinding.

Outcome evaluations were performed by an independent team of assessors recruited from hospitals unaffiliated with the study sites. These assessors were blinded to group assignments, and all assessment forms were coded with participant identification numbers without indicating group allocation. Furthermore, statistical analyses were conducted by a biostatistician who remained blinded to group assignments until all primary analyses were completed. The master list of participant allocations was securely maintained by a research coordinator in a password-protected file and was not accessed until after data collection and analysis had concluded.

1. Assessment Phase

The assessment phase aimed to establish baseline measurements of nurses’ knowledge, clinical competencies, and adherence to the PNE Model principles using four validated instruments: the Participant Characteristics Questionnaire, Nurses’ Knowledge Assessment Questionnaire, Observational Checklist for Nursing Practice, and Pediatric Nursing Excellence (PNE) Model Questionnaire. These instruments measured contextual data, nursing knowledge, practical skills, and adherence to PNE Model principles. Detailed descriptions of each tool, including validation methods, reliability scores, and scoring systems, are provided in Section 2.6. All assessments were conducted in a quiet, private setting to ensure unbiased responses. Each nurse was individually evaluated in a standardized environment, with only the assessor and the participant present to reduce external influences and enhance focus. The outcome assessors, blinded to group assignments, conducted these evaluations to maintain objectivity, reduce observer bias, and ensure that all data collection was carried out consistently. Both the intervention and control groups underwent identical baseline assessments, ensuring uniformity and consistency in the evaluation process. Assessors adhered to a strict protocol to avoid deviations during data collection, and assessments were scheduled at times that minimized interference with the nurses’ regular duties, further reducing potential bias.

2. Planning Phase

The planning phase focused on preparing the PNE Model intervention and ensuring the readiness of the facilitators and materials. A comprehensive intervention manual was developed, outlining both theoretical and practical aspects of the PNE Model:

Theoretical Foundations: Core principles of pediatric orthopedic care and the importance of nursing excellence.

Practical Applications: Step-by-step procedures for implementing the PNE Model in clinical settings.

Training materials were developed to ensure consistent delivery, including handouts summarizing key concepts, audiovisual aids such as PowerPoint presentations and instructional videos, and case studies illustrating real-life scenarios in pediatric orthopedic care. The facilitators underwent rigorous training to ensure standardized delivery across sessions. The facilitators were all experienced pediatric nursing educators, prepared to address both theoretical and practical dimensions of the PNE Model using adult learning strategies to foster active participation.

3. Implementation Phase

The implementation phase consisted of two parallel training programs conducted over four weeks from March to April 2024. Both programs were structured to ensure methodological rigor and maintain the integrity of the double-blind design.

3.1. Intervention Group Training

The PNE Model intervention consisted of six structured training sessions focusing on essential domains of pediatric orthopedic care. Each session included theoretical instruction, simulation-based learning, and practical applications of family-centered and evidence-based care principles, enabling nurses to translate theory into clinical practice effectively. These sessions were designed to accommodate clinical shifts and minimize disruption to patient care. Each session was facilitated by pediatric nursing specialists who underwent standardized training in PNE Model delivery. The intervention curriculum was structured as follows:

Session 1: Introduction to Pediatric Orthopedic Surgeries Comprehensive overview of pediatric orthopedic conditions, incorporating evidence-based classifications, contemporary etiological frameworks, and current diagnostic criteria. Interactive discussions facilitated experiential learning and misconception clarification.

Session 2: Diagnostics and Complications of Orthopedic Surgeries: Advanced exploration of diagnostic modalities, including radiological investigations (X-rays, MRI, CT scans) and their clinical implications. Evidence-based case studies were utilized to demonstrate diagnostic reasoning and complication management strategies.

Session 3: Preoperative Care and Transfer Procedures: Integration of psychological preparation protocols, informed consent processes, and evidence-based transfer guidelines. Simulation-based learning and role-playing scenarios enhanced skill acquisition and clinical competency.

Session 4: Postoperative Care and Clinical Management: Implementation of systematic vital signs monitoring, evidence-based pain assessment tools, and contemporary wound care protocols. High-fidelity simulation exercises facilitated the practical application of theoretical principles.

Session 5: Patient Education and Discharge Planning: Development of family-centered education strategies and individualized discharge planning protocols. Practical application through simulated scenarios enhanced competency in caregiver education and discharge preparation.

Session 6: Clinical Integration of PNE Model Principles: Synthesis of PNE Model principles through practical implementation strategies and clinical scenarios. Group activities and reflective practice sessions facilitated the development of evidence-based action plans.

3.2. Control Group Training

The control group participated in a matched educational program consisting of six 90 min sessions delivered by hospital educators. These sessions were systematically structured to address core nursing competencies:

Session 1: Clinical Safety and Infection Control: Evidence-based infection prevention protocols and institutional safety guidelines.

Session 2: Clinical Documentation and Quality Standards: Contemporary documentation practices and quality metric compliance.

Session 3: Patient Assessment and Care Planning: Systematic assessment techniques and care plan development.

Session 4: Professional Communication: Therapeutic communication strategies and interprofessional collaboration.

Session 5: Quality Management: Risk assessment protocols and incident management procedures.

Session 6: Professional Development: Evidence-based practice principles and continuing education frameworks.

4. Evaluation Phase

The evaluation phase involved post-intervention assessments conducted at four key time points: immediately after the intervention and at 1, 3, and 6 months post-intervention. The follow-up intervals immediately post-intervention and at 1, 3, and 6 months were strategically selected based on established research methodologies in nursing education. The immediate post-intervention assessment evaluates the direct impact of the training. The one-month follow-up assesses short-term knowledge retention, aligning with studies indicating that significant knowledge decay can occur within weeks if not reinforced [63]. The three-month interval examines intermediate retention, considering findings that clinical skill decay can begin within three months without ongoing practice [10]. The six-month follow-up evaluates long-term retention and the sustainability of practice changes, as the literature suggests that substantial skill deterioration may occur without reinforcement [66]. These intervals are consistent with prior research demonstrating that changes in clinical practice patterns often emerge within three to six months following educational interventions [67]. The same instruments from the assessment phase (Tools I, II, and III) were used to ensure consistency in measurement. Outcome assessors, who remained blinded to group assignments, conducted these evaluations to maintain objectivity. Practical skills were evaluated using the Observational Checklist during routine clinical activities, ensuring minimal interference with the normal workflow. Inter-rater reliability was maintained through standardized observation protocols and ongoing assessor training.

#### 2.9.2. Quality Assurance and Ethical Considerations

Throughout the study, quality assurance measures were implemented to ensure the integrity and reliability of data. The intervention protocol was strictly followed, with regular meetings with facilitators to address challenges and ensure consistency. Fidelity checks were conducted to confirm that the intervention was delivered as intended. Participant confidentiality was safeguarded by assigning unique codes to each nurse and securely storing all data. The study was registered with ClinicalTrials.gov (Identifier: NCT06624007). Written informed consent was obtained from all participants, who were informed of the study’s purpose, procedures, potential risks, and benefits. The informed consent process included detailed explanations using accessible language to ensure participants’ full comprehension. Participation was voluntary, and nurses were assured they could withdraw at any time without repercussions.

### 2.10. Statistical Analysis

Data were analyzed using IBM SPSS Statistics 27.0, with statistical significance set at *p* < 0.05. Descriptive statistics (mean, standard deviation, frequencies, and percentages) were calculated for demographic and baseline characteristics. To ensure group comparability at baseline, independent *t*-tests were used for continuous variables, and chi-square tests were applied for categorical variables. Repeated measures ANOVA was used to evaluate changes in knowledge and practice scores within groups over time, including assessments immediately post-intervention and at 1, 3, and 6 months. The Greenhouse–Geisser correction was applied when the assumption of sphericity was violated. Effect sizes were calculated using Cohen’s d, interpreted as small (0.2), medium (0.5), or large (0.8). Pearson correlation coefficients were used to explore relationships between changes in knowledge and practice scores and variables such as years of experience, prior training in pediatric orthopedic care, and adherence to the PNE Model.

**Figure 3:**
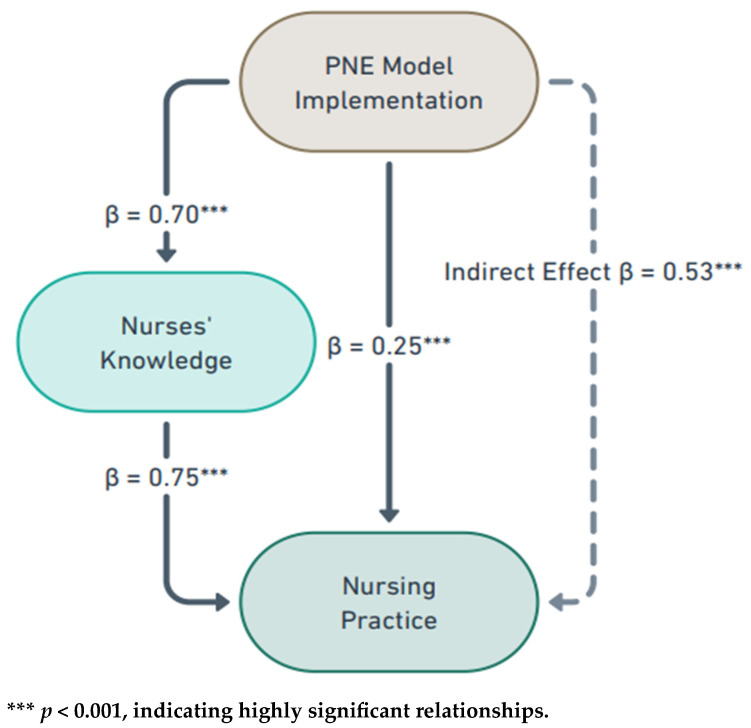
Path diagram illustrating the effects of PNE Model implementation on nurses’ knowledge and nursing practice.

Multivariate linear regression was conducted to examine factors influencing post-intervention knowledge and practice scores, adjusting for potential confounders such as age, years of experience, educational qualifications, prior training in pediatric orthopedic care, and baseline scores. Standardized coefficients (β) and 95% confidence intervals (CI) were reported to quantify the strength and direction of these relationships. Path analysis using structural equation modeling was employed to evaluate the direct and indirect effects of the PNE Model on nursing practice, with knowledge acquisition assessed as a mediator. Model fit was evaluated using the chi-square test (χ^2^), Comparative Fit Index (CFI ≥ 0.95), Root Mean Square Error of Approximation (RMSEA ≤ 0.06), and Standardized Root Mean Square Residual (SRMR ≤ 0.08), ensuring the robustness of the analytical model. The results of these analyses are detailed in Figure 3. To assess the sustainability of improvements in knowledge and practice over time, paired *t*-tests were used to compare scores at different follow-up intervals. Multiple imputation techniques were applied to maintain the validity of the results. A blinded statistician performed all statistical analyses to reduce bias and enhance objectivity in data interpretation.

## 3. Results

This section presents the study’s findings, highlighting the changes in knowledge and practice scores among nurses in the intervention and control groups, alongside detailed analyses of adherence to the Pediatric Nursing Excellence (PNE) model and the retention of improvements over time. The results are presented in accordance with the study’s timeline, including baseline assessments and post-intervention follow-ups at 1, 3, and 6 months. Statistical analyses, including effect sizes and *p*-values, are used to determine the significance and practical implications of the findings. Each table provides a comprehensive overview of the data, with accompanying comments to interpret the results in relation to the study’s objectives.

Table 1 presents the demographic characteristics of the nurse participants. Statistical comparisons using *t*-tests for continuous variables and chi-square tests for categorical variables confirm that the intervention and control groups were comparable across all assessed characteristics, indicating successful randomization. For example, the mean age was 33.76 ± 9.97 years in the intervention group and 35.64 ± 10.85 years in the control group, with no significant difference (t = −0.917, *p* = 0.361). Similarly, the majority of participants in both groups were female (88% intervention vs. 92% control, χ^2^ = 0.444, *p* = 0.505) and held diplomas from a Technical Institute of Nursing (80% intervention vs. 88% control, χ^2^ = 1.422, *p* = 0.491). Years of nursing experience were comparable, with a mean of 13.94 ± 9.74 years in the intervention group and 15.70 ± 10.69 years in the control group (t = −0.873, *p* = 0.385). Prior training in pediatric orthopedic care was low across both groups (26% intervention vs. 20% control), with no significant difference (χ^2^ = 0.476, *p* = 0.490), indicating a general need for specialized education in this area. Among nurses who reported prior training (intervention group: *n* = 13; control group: *n* = 10), the type and duration of training varied. Most (*n* = 15) participated in hospital-based workshops lasting 3 days, focused on basic principles of pediatric orthopedic care. Five nurses completed 1-week professional development courses offered by nursing associations, and three nurses attended 3-month specialized certification programs in pediatric orthopedic nursing. These training sessions occurred 6 months to 3 years prior to the study, with a median duration of 5 days. Notably, these programs were neither as comprehensive nor as structured as the PNE Model intervention. This detailed analysis underscores the inconsistent and limited nature of prior training and highlights the need for more standardized, comprehensive training approaches like the PNE Model.

Table 2 illustrates the mean knowledge and practice scores for both groups at baseline and at subsequent time points. At baseline, there were no significant differences between the intervention and control groups in knowledge (7.98 ± 5.4 vs. 8.26 ± 5.48, respectively; *p* = 0.673) or practice scores (40.12 ± 14.2 vs. 39.90 ± 14.5; *p* = 0.900), with a negligible effect size (Cohen’s d = 0.05). Immediately post-intervention, the intervention group exhibited a significant increase in knowledge scores (20.62 ± 6.7) compared to the control group (8.16 ± 5.5), with a large effect size (Cohen’s d = 1.82; *p* < 0.001). This improvement was sustained over time, with the intervention group maintaining higher knowledge scores at the 1-, 3-, and 6-month follow-ups (*p* < 0.001 at all time points). Similarly, practice scores in the intervention group increased significantly immediately post-intervention (62.28 ± 4.1) compared to the control group (40.06 ± 14.7), with a substantial effect size (Cohen’s d > 1.5; *p* < 0.001). The gains in practice were also maintained over the 6-month period. The control group’s scores remained relatively unchanged throughout the study. The consistent statistical significance and large effect sizes indicate a robust impact of the PNE Model on both knowledge and practice.

Table 3 provides a granular analysis of post-intervention scores by specific domains and content areas. In all practice domains, the intervention group outperformed the control group significantly. For preoperative care, the intervention group scored 11.06 ± 1.8 compared to 7.00 ± 4.0 in the control group (Cohen’s d = 1.18; *p* < 0.001). Transfer care scores were also higher in the intervention group (4.74 ± 0.52 vs. 3.16 ± 1.5; Cohen’s d = 1.22; *p* < 0.001). In postoperative care, the most substantial difference was observed, with the intervention group scoring 39.10 ± 3.27 versus 25.46 ± 9.54 in the control group (Cohen’s d = 1.87; *p* < 0.001). Health education and discharge planning scores similarly favored the intervention group (7.38 ± 0.75 vs. 4.32 ± 2.46; Cohen’s d = 1.64; *p* < 0.001).

For knowledge content areas, the intervention group consistently achieved higher scores. For instance, knowledge of definitions of orthopedic conditions was significantly greater in the intervention group (8.4 ± 1.2) compared to the control group (5.7 ± 1.4), with a large effect size (Cohen’s d = 1.72; *p* < 0.001). Similar significant differences and large effect sizes were observed across all knowledge areas assessed. These results underscore the PNE Model’s comprehensive effectiveness in enhancing specific knowledge and practical competencies.

Table 4 explores the relationships between improvements in knowledge and practice scores and other variables within the intervention group. There was a strong positive correlation between changes in knowledge and practice scores (r = 0.724; *p* < 0.001), indicating that increases in knowledge were associated with improvements in practice. Years of nursing experience correlated moderately with both knowledge (r = 0.320; *p* < 0.01) and practice improvements (r = 0.415; *p* < 0.01), suggesting that more experienced nurses benefited slightly more from the intervention. Prior training in pediatric orthopedic care showed a moderate positive correlation with knowledge (r = 0.424; *p* < 0.01) and practice improvements (r = 0.471; *p* < 0.01), indicating that previous exposure to the subject matter may enhance the effectiveness of the PNE Model. Adherence to the PNE Model principles also correlated positively with knowledge (r = 0.625; *p* < 0.001) and practice improvements (r = 0.671; *p* < 0.001), reinforcing the significance of model fidelity in achieving desired outcomes.

Table 5 demonstrates a statistically significant improvement in adherence to the Pediatric Nursing Excellence (PNE) Model among the intervention group. Mean adherence scores increased from 30.12 ± 6.18 at baseline to 45.34 ± 5.94 post-intervention (*p* < 0.001), with a large effect size (Cohen’s d = 2.53). Notably, the proportion of nurses achieving high excellence levels increased dramatically from 8% to 82%. These results provide robust evidence for the efficacy of the PNE Model in enhancing professional excellence in pediatric orthopedic nursing. However, several methodological considerations warrant attention, including potential Hawthorne and ceiling effects, as well as the long-term sustainability of these improvements. Future research should aim to elucidate the specific mechanisms underlying these substantial gains and assess their persistence over time, thereby strengthening the evidence base for the PNE Model’s impact on nursing practice.

Table 6 assesses the sustainability of the intervention’s effects within the intervention group over six months. Knowledge scores remained high immediately post-intervention (20.62 ± 6.7) and showed only a slight, non-significant decline over time (19.35 ± 7.2 at six months; *p* > 0.05). Similarly, practice scores were sustained from immediately post-intervention (62.28 ± 4.1) to 6 months (60.76 ± 4.9; *p* > 0.05). The lack of significant decline in both knowledge and practice scores indicates that the improvements achieved through the PNE Model were retained over time, demonstrating the intervention’s lasting impact.

Table 7 presents the results of a multivariate analysis examining factors influencing post-intervention knowledge and practice scores in pediatric orthopedic nursing care. This analysis provides insights into the relative impact of various factors while controlling for potential confounders. The intervention remained the strongest predictor of improved scores, with significantly higher knowledge (β = 12.46, 95% CI: 11.78, 13.14, *p* < 0.001) and practice scores (β = 22.22, 95% CI: 21.03, 23.41, *p* < 0.001) in the intervention group compared to the control group. This robust effect persisted even after adjusting for other variables, reinforcing the effectiveness of the Pediatric Nursing Excellence (PNE) Model.

Prior training in pediatric orthopedic care also emerged as a significant predictor of both knowledge (β = 0.73, 95% CI: 0.03, 1.43, *p* = 0.041) and practice scores (β = 1.28, 95% CI: 0.06, 2.50, *p* = 0.039). This suggests that nurses with previous specialized training were able to build upon their existing knowledge and skills more effectively.

Baseline scores were significant predictors of post-intervention scores for both knowledge (β = 0.42, 95% CI: 0.35, 0.49, *p* < 0.001) and practice (β = 0.38, 95% CI: 0.31, 0.45, *p* < 0.001), indicating that nurses with higher initial scores tended to maintain their advantage throughout the intervention. Interestingly, age, years of experience, and educational qualifications did not show statistically significant independent effects on post-intervention scores when controlling for other factors. This suggests that the PNE Model was equally effective across different age groups, experience levels, and educational backgrounds.

These results confirm the robust effect of the PNE Model intervention on improving nurses’ knowledge and practice in pediatric orthopedic care, even when accounting for other potentially influential factors. The findings also highlight the importance of prior specialized training and baseline competency levels in maximizing the benefits of professional development interventions. The lack of significant effects for demographic variables suggests that the PNE Model can be effectively implemented across diverse nursing populations, potentially leading to standardized improvements in pediatric orthopedic nursing care.

Model Fit Indices:

- Chi-Square (χ^2^): The chi-square statistic was non-significant (χ^2^ = 1.85, degrees of freedom [df] = 1, *p* = 0.174), suggesting that the model fits the observed data well.
- Comparative Fit Index (CFI): The CFI was 0.998, exceeding the recommended threshold of 0.95 for a good-fitting model, indicating excellent model fit.
- Root Mean Square Error of Approximation (RMSEA): The RMSEA was 0.045, below the acceptable limit of 0.06, further supporting the model’s adequacy.
- Standardized Root Mean Square Residual (SRMR): The SRMR was 0.012, well under the recommended maximum of 0.08, confirming the model’s excellent fit to the data.

Table 8 presents the path analysis results, highlighting the influence of the Pediatric Nursing Excellence (PNE) Model on nurses’ knowledge and practice through structural equation modeling. The PNE Model significantly positively affected nurses’ knowledge (β = 0.70, *p* < 0.001), indicating the intervention’s success in enhancing understanding of pediatric orthopedic care. This improved knowledge strongly impacted nursing practice (β = 0.75, *p* < 0.001), demonstrating a clear link between increased knowledge and improved clinical skills. Additionally, the PNE Model significantly influenced nursing practice independent of knowledge (β = 0.25, *p* < 0.001), suggesting that factors such as engagement and adherence to excellence principles also contributed to practice improvements. The indirect effect of the PNE Model on practice, mediated by knowledge, was significant (β = 0.53, *p* < 0.001), underscoring the central role of knowledge as a mediator.

Overall, the total effect of the PNE Model on nursing practice was substantial (β = 0.78), affirming the intervention’s comprehensive impact. The model fit indices, including CFI = 0.998, RMSEA = 0.045, and SRMR = 0.012, confirmed the robustness of the model. The path analysis indicates that the PNE Model significantly enhances nursing practice by improving knowledge and contributing through other mechanisms. These findings validate the PNE Model as an effective intervention for advancing nursing competencies in pediatric orthopedic care, consistent with the model’s theoretical framework. Figure 3 visually represents these findings, reinforcing the direct and indirect pathways through which the PNE Model affects nursing practice.

## 4. Discussion

This randomized controlled trial evaluated the effectiveness of the PNE Model in enhancing nurses’ knowledge and practice in pediatric orthopedic surgery care. The findings demonstrate that implementing the PNE Model significantly improved both the theoretical understanding and practical competencies of nurses in the intervention group compared to the control group, with these improvements sustained over six months.

### 4.1. Enhancement of Nurses’ Knowledge and Practice

The significant improvement in nurses’ knowledge aligns with recent systematic reviews of nursing educational interventions. For instance, Sapri et al. (2022) found that structured educational programs incorporating simulation and mentorship components showed similar effectiveness in improving clinical knowledge, with sustained effects over time [68]. Our findings extend this work by demonstrating particular effectiveness in the specialized context of pediatric orthopedic care. Similar to Moore et al.’s (2023) multi-center study of nursing excellence programs, our results suggest that theory-based educational interventions can produce lasting improvements in both knowledge and practice [69]. This improvement aligns with the primary Hypothesis (H1) that nurses trained using the PNE Model would demonstrate significantly higher knowledge scores post-intervention. These findings are consistent with prior studies emphasizing the impact of structured educational interventions on nursing knowledge. For instance, Valdivia et al. (2022) reported that specialized training programs significantly enhanced nurses’ understanding of pediatric orthopedic care principles [70]. Similarly, the practice scores of the intervention group improved markedly across all domains: preoperative care, transfer care, postoperative care, and health education immediately post-intervention and were maintained over six months. This supports the second Hypothesis (H2) that the intervention group would exhibit superior clinical practice performance. The sustained nature of these improvements suggests that the PNE Model not only facilitates immediate skill acquisition but also promotes long-term retention, corroborating the findings of Doleman (2022), who highlighted the effectiveness of continuous professional development and mentorship in nursing practice [71].

### 4.2. Correlation Between Knowledge and Practice

Analysis of the relationship between knowledge acquisition and clinical practice demonstrated significant positive correlations, validating our third hypothesis. This finding aligned with Liu et al.’s (2023) meta-analysis of 42 nursing education programs, which established that integrated theoretical-practical approaches yielded superior clinical outcomes compared to traditional pedagogical methods [72]. Fontaine et al.’s (2024) systematic review of 28 nursing excellence programs further corroborated these results, emphasizing that structured implementation frameworks significantly enhanced the translation of theoretical knowledge into clinical competence [53]. The successful knowledge-practice integration observed in our study exemplified Benner’s Novice to Expert theory [40], particularly regarding the role of experiential learning in developing clinical expertise. This theoretical framework, as validated by recent empirical studies [73], provided a robust explanation for how the PNE Model facilitated the transformation of theoretical understanding into enhanced clinical performance. The sustained improvement in practice scores suggested that the model effectively bridged the theory-practice gap, a persistent challenge in nursing education identified in contemporary literature [74].

### 4.3. Adherence to the PNE Model and Excellence in Practice

The implementation of the PNE Model significantly improved nurses’ adherence to principles of professional excellence. These improvements reflect the intervention’s success in instilling core nursing values such as engagement, ethical decision-making, and continuous improvement, which are fundamental for delivering high-quality care. Existing literature corroborates the findings of this study. For instance, Naz et al. (2020) highlighted that a supportive work environment characterized by professional commitment and structured development programs is essential for nursing excellence [75]. Similarly, Brown et al. (2020) demonstrated that professional education combined with mentorship and regular feedback enhances adherence to excellence-focused practices, leading to improved patient outcomes and increased professional satisfaction among nurses [76]. The PNE Model aligns with these principles, emphasizing structured learning and reflective practice to foster a culture of professional development. This study’s results align with Benner’s Novice to Expert theory, which underscores the importance of experiential learning and structured guidance in advancing nursing competency [40]. The PNE Model’s structured framework allows nurses to bridge the gap between theoretical knowledge and clinical application, supporting the development of higher-order skills and professional identity [14,30,37,38,77].

However, some limitations should be noted. The Hawthorne effect could have influenced the nurses’ behaviors, as their awareness of being studied might have enhanced their short-term adherence to excellence practices. Furthermore, the high level of excellence achieved by many participants post-intervention suggests a possible ceiling effect, potentially masking further improvements. These factors highlight the need for ongoing interventions to sustain and build upon these achievements. Future research should focus on the long-term sustainability of the improvements observed in this study. Investigating the specific mechanisms by which the PNE Model influences adherence to excellence principles, such as the roles of mentorship, organizational support, and peer collaboration, would provide valuable insights. Additionally, expanding the application of the PNE Model to diverse clinical settings, such as neonatal intensive care or critical care, could further demonstrate its versatility and broader impact. In conclusion, the PNE Model is an effective intervention for enhancing adherence to professional excellence principles in pediatric nursing. Combining structured training with continuous development and reflective practice addresses gaps in traditional nursing education and supports nurses’ professional growth. These findings contribute to the growing body of evidence on the importance of integrating theoretical frameworks with practical interventions to achieve nursing excellence.

### 4.4. Discussion of Specific Domains and Content Areas

A detailed examination of practice domains and knowledge content areas demonstrated marked improvements attributable to the PNE Model intervention. The findings highlight the model’s ability to enhance critical preoperative and postoperative care competencies, reinforcing its value as a structured approach to professional development in pediatric nursing. In preoperative care, the PNE Model effectively supported nurses in developing the skills needed to prepare pediatric patients for surgery, a crucial step in mitigating preoperative anxiety and optimizing surgical outcomes. Research has consistently shown that anxiety management and effective preparation before surgery are key to improving postoperative recovery and reducing the psychological burden on young patients and their families [78,79,80]. The PNE Model incorporates theoretical knowledge with hands-on application, enabling nurses to build confidence and proficiency in areas such as patient education, procedural explanations, and parental involvement, all of which are critical for holistic preoperative care.

The most substantial improvements were observed in postoperative care, where the intervention group demonstrated enhanced competencies in monitoring vital signs, managing pain, preventing complications, and providing effective discharge planning. These competencies are particularly important, as they directly influence patient recovery, complication rates, and overall satisfaction with care. Studies by Köse Tamer et al. (2020) [81] and Kwame et al. (2019) [82] have highlighted that well-trained nurses who adhere to structured postoperative care protocols are better equipped to identify early signs of complications, provide effective pain management, and ensure seamless transitions from hospital to home care. The PNE Model’s emphasis on clinical checklists, pain management strategies, and patient education aligns with these best practices and underscores its effectiveness in standardizing high-quality care. The intervention’s impact on knowledge content areas, such as understanding the principles of orthopedic care, postoperative monitoring, and health education, further validates the model’s theoretical framework. The integration of experiential learning with reflective practice is central to fostering the depth of knowledge necessary for advanced clinical decision-making. Future research should explore how the PNE Model’s principles can be adapted for other pediatric specialties and investigate the long-term retention of skills and knowledge gained through this intervention. Additionally, examining patient outcomes, such as reduced complications, shorter hospital stays, and increased family satisfaction, would provide further evidence of the model’s efficacy.

### 4.5. Comparison with Contradictory Findings

While our study demonstrated the positive impact of the PNE Model in improving nursing knowledge and practice, it is important to contextualize these findings within the broader body of literature, including studies with contrasting results. Peng et al. (2023) highlighted that high patient-to-nurse ratios and inconsistent clinical practice standards can hinder the effectiveness of structured nursing education programs [83]. These systemic issues, often beyond the control of individual interventions, can limit nurses’ ability to fully implement learned competencies in practice, thereby attenuating the potential benefits of such models. In our study, these factors were meticulously controlled across both intervention and control groups. For instance, nursing workloads and practice environments were standardized to ensure a consistent baseline for evaluating the PNE Model’s effectiveness. This controlled environment likely contributed to the consistent positive outcomes observed in our results, suggesting that the PNE Model has robust potential when implemented in well-regulated settings.

However, the findings underscore the importance of addressing broader systemic challenges such as staffing levels and standardization of care protocols. Several studies emphasize that educational interventions alone may not sustain improvements unless supported by institutional policies that ensure optimal nurse-to-patient ratios and adherence to evidence-based practices. For example, Dall’Ora et al. (2022) demonstrated that higher nurse staffing levels were directly associated with improved patient outcomes, even when controlling for other variables [84]. Similarly, McLachlan et al. (2020) reported that variability in care protocols diminished the effectiveness of standardized nursing models in real-world settings, reinforcing the need for systemic alignment [85]. These considerations highlight the critical role of health system infrastructure in enhancing the efficacy of educational interventions like the PNE Model. Addressing organizational barriers, fostering a supportive practice environment, and ensuring adequate staffing are necessary to maximize the potential of such frameworks. Future research should explore the interaction between nursing education interventions and systemic factors to understand better how to optimize their implementation and impact in diverse clinical settings.

### 4.6. Path Analysis and Model Fit

The path analysis clearly demonstrated the mechanisms through which the Pediatric Nursing Excellence (PNE) Model influenced the development of nursing practice. The direct and indirect effects observed validated the theoretical foundation of the intervention. The mediating role of knowledge enhancement in improving clinical skills aligns with findings by Mlambo et al. (2021), which emphasized the importance of professional development programs in fostering both theoretical understanding and practical application in nursing practice [37]. The strong model fit indices reinforced the structural validity of the PNE Model. This validation builds on frameworks such as Benner’s Novice to Expert theory, which emphasizes the progression of nursing competence through experiential learning, and Duffy’s Quality-Caring Model, which highlights the role of structured interventions in integrating theory and practice in clinical environments [86]. These theoretical frameworks provided a robust basis for interpreting how the PNE Model bridges the gap between conceptual understanding and clinical application in pediatric orthopedic care.

Moreover, the results align with the work of Chesak et al. (2022), who proposed that practice-based models in nursing science can effectively integrate knowledge acquisition with skill development [36]. Similarly, the findings contribute to the broader discourse on nursing education and specialized care, echoing Portela Dos Santos et al.’s (2022) systematic review on professional excellence, which emphasized the critical role of structured education in promoting sustained improvements in clinical practice [55]. By establishing empirical support for theoretical pathways in specialized pediatric care, this analysis extends the contemporary understanding of nursing excellence development. Future studies could explore the applicability of these findings across different nursing specialties and healthcare contexts, further strengthening the evidence base for the PNE Model as a transformative framework in nursing education and practice.

### 4.7. Limitations

Several limitations of this study should be acknowledged. First, the sample was drawn from two hospitals within a single geographic region in Egypt, which may limit the generalizability of the findings to other settings or countries with different healthcare systems. Second, the study did not assess patient outcomes directly, such as complication rates or patient satisfaction, which would provide a more comprehensive evaluation of the PNE Model’s impact. This omission limits the study’s objectivity in linking nursing competency improvements to measurable patient outcomes. Including patient-centered metrics such as reduced recovery times, complication rates, or enhanced satisfaction levels would provide a more robust validation of the intervention’s effectiveness. Third, reliance on self-reported measures and observations could introduce bias, although efforts were made to minimize this through blinding and standardized assessment tools.

Another limitation of this study is the absence of training or evaluation regarding the administration of high-risk medications, such as morphine derivatives, which are frequently used in pediatric orthopedic care. High-alert medications pose significant safety risks if not handled appropriately, and their omission from the study limits the comprehensiveness of the intervention. Including such components in future studies would provide a more holistic approach to improving pediatric nursing competencies, as supported by recent findings on the need for decision-support systems to guide high-alert medication administration [87].

### 4.8. Practical Implications

The findings have significant practical implications for nursing education and clinical practice. Incorporating repeated training sessions and the use of reminder materials, such as ward posters or practical assignments for nurses, could enhance the sustainability of training benefits. These strategies align with evidence demonstrating the efficacy of continuous reinforcement to maintain competency levels in clinical practice. Implementing the PNE Model can serve as a blueprint for developing specialized training programs that enhance nurses’ competencies in pediatric orthopedic care. Hospitals and educational institutions should consider integrating such models into their curricula and professional development initiatives. Moreover, emphasizing continuous professional development and mentorship can lead to sustained improvements in practice, ultimately enhancing patient care quality and safety.

### 4.9. Future Directions

Future research should explore the long-term effects of the PNE Model on patient outcomes, including recovery times, complication rates, and satisfaction levels among pediatric patients and their families. Additionally, future implementations should integrate ongoing refresher courses and reinforcement tools, such as educational posters and interactive assignments, to evaluate how these additions influence both nursing practice and patient outcomes over time. These approaches could also investigate how to mitigate the Hawthorne effect and ensure consistent application of learned principles across varying clinical environments. Additionally, studies could investigate the model’s applicability in other specialized areas of pediatric care, such as oncology or critical care, to determine its broader utility. Expanding the research to include multiple geographic regions and diverse healthcare settings would also enhance the generalizability of the findings.

## 5. Conclusions

The PNE Model is an effective intervention for improving nurses’ knowledge and practice in pediatric orthopedic surgery care. By addressing the specific challenges of pediatric orthopedic nursing through a structured and theory-based approach, the model facilitates significant and sustained enhancements in both theoretical understanding and clinical competencies. These improvements are directly correlated with adherence to the model’s principles, emphasizing the importance of engagement, continuous improvement, and holistic care in achieving nursing excellence. Implementing the PNE Model can contribute to higher standards of care in pediatric orthopedics, ultimately leading to better patient outcomes and satisfaction.

## Figures and Tables

**Table 1:** Demographic characteristics of nurse participants in the intervention and control groups.

Personal Characteristics	Intervention Group (*n* = 50)	Control Group (*n* = 50)	Test Statistic	*p*-Value
Age (Years)			t = −0.917	0.361
<20	2 (4%)	2 (4%)		
20–<30	16 (32%)	16 (32%)		
30–<40	15 (30%)	14 (28%)		
≥40	17 (34%)	18 (36%)		
Mean ± SD	33.76 ± 9.97	35.64 ± 10.85		
Gender			χ^2^ = 0.444	0.505
Male	6 (12%)	4 (8%)		
Female	44 (88%)	46 (92%)		
Educational Qualifications			χ^2^ = 1.422	0.491
Technical institute of nursing	40 (80%)	44 (88%)		
Bachelor’s degree in nursing	8 (16%)	5 (10%)		
Postgraduate Studies	2 (4%)	1 (2%)		
Years of Experience			t = −0.873	0.385
<5 years	10 (20%)	8 (16%)		
5–<10 years	10 (20%)	11 (22%)		
≥10 years	30 (60%)	31 (62%)		
Training in Pediatric Orthopedic Care			χ^2^ = 0.476	0.490
Yes	13 (26%)	10 (20%)		
No	37 (74%)	40 (80%)		

SD = standard deviation. *t*-tests were used for age and experience, and chi-square tests were used for categorical variables. All the *p*-values were >0.05, indicating no significant differences between groups. Prior training in pediatric orthopedic care includes hospital-based workshops (3 days), professional development courses (1 week), and specialized certification programs (3 months), completed 6 months to 3 years prior to the study.

**Table 2:** Changes in knowledge and practice scores over time in the intervention and control groups.

Time Point	Intervention Group (*n* = 50)	Control Group (*n* = 50)	Effect Size (Cohen’s d)	*p*-Value (Knowledge)	*p*-Value (Practice)
Baseline					
Knowledge	7.98 ± 5.4	8.26 ± 5.48	0.05	0.673	0.900
Practice	40.12 ± 14.2	39.90 ± 14.5			
Immediately post-intervention					
Knowledge	20.62 ± 6.7	8.16 ± 5.5	1.82	<0.001	<0.001
Practice	62.28 ± 4.1	40.06 ± 14.7			
1-Month Follow-Up					
Knowledge	20.15 ± 6.9	8.10 ± 5.6	1.78	<0.001	<0.001
Practice:	61.95 ± 4.4	40.05 ± 14.9			
3-Month Follow-Up					
Knowledge	19.72 ± 7.1	8.04 ± 5.7	1.70	<0.001	<0.001
Practice	61.23 ± 4.7	39.99 ± 15.0			
6-Month Follow-Up					
Knowledge	19.35 ± 7.2	8.00 ± 5.8	1.61	<0.001	<0.001
Practice	60.76 ± 4.9	39.95 ± 15.1			

**Table 3:** Detailed knowledge and practice scores by domain and content area at post-intervention.

Domain/Content Area	Intervention Group (*n* = 50)	Control Group (*n* = 50)	Effect Size (Cohen’s d)	*p*-Value
Preoperative Care (Practice)	11.06 ± 1.8	7.00 ± 4.0	1.18	<0.001
Transfer Care (Practice)	4.74 ± 0.52	3.16 ± 1.5	1.22	<0.001
Postoperative Care (Practice)	39.10 ± 3.27	25.46 ± 9.54	1.87	<0.001
Health Education and Discharge (Practice)	7.38 ± 0.75	4.32 ± 2.46	1.64	<0.001
Definitions of Orthopedic Conditions (Knowledge)	8.4 ± 1.2	5.7 ± 1.4	1.72	<0.001
Causes of Orthopedic Conditions (Knowledge)	7.8 ± 1.3	5.9 ± 1.3	1.46	<0.001
Types of Orthopedic Surgeries (Knowledge)	7.6 ± 1.4	5.6 ± 1.5	1.38	<0.001
Diagnostics (Knowledge)	6.9 ± 1.2	5.4 ± 1.6	1.20	<0.001
Complications (Knowledge)	7.1 ± 1.3	5.3 ± 1.7	1.19	<0.001
Preoperative Care Principles (Knowledge)	7.4 ± 1.5	5.5 ± 1.4	1.28	<0.001
Postoperative Care Principles (Knowledge)	8.1 ± 1.2	6.0 ± 1.3	1.45	<0.001

Values are presented as mean ± standard deviation. Effect size is calculated using Cohen’s d. *p*-values are derived from independent *t*-tests comparing intervention and control groups. Practice domains (Preoperative Care, Transfer Care, Postoperative Care, Health Education and Discharge) are scored on the Observational Checklist for Nursing Practice. Knowledge content areas are scored on the Nurses’ Knowledge Assessment Questionnaire.

**Table 4:** Correlation between knowledge, practice improvements, and other variables in the intervention group.

Variable	Knowledge Score Improvement (r)	Practice Score Improvement (r)	*p*-Value
Years of Nursing Experience	0.320	0.415	<0.01
Prior Training in Pediatric Orthopedic Care	0.424	0.471	<0.01
Change in Knowledge Scores	-	0.724	<0.001
Change in Practice Scores	0.724	-	<0.001
Adherence to the PNE Model	0.625	0.671	<0.001

r = Pearson correlation coefficient. *p*-values indicate the statistical significance of the correlations. PNE = Pediatric Nursing Excellence. Improvements and changes in scores refer to the difference between post-intervention and baseline measurements. Adherence to the PNE Model was measured using the PNE Model Questionnaire.

**Table 5:** Adherence to Pediatric Nursing Excellence (PNE) Model principles in the intervention group (*n* = 50).

Variable	Baseline (*n* = 50)	Post-Intervention (*n* = 50)	Effect Size (Cohen’s d)	*p*-Value
Mean PNE Model Adherence Score	30.12 ± 6.18	45.34 ± 5.94	2.53	<0.001
Excellence Level:				<0.001
Low	24 (48%)	3 (6%)	-	-
Moderate	22 (44%)	6 (12%)	-	-
High	4 (8%)	41 (82%)	-	-

PNE = Pediatric Nursing Excellence. Adherence scores range from 0 to 56, with higher scores indicating greater adherence. Excellence levels: low (<28), moderate (28–41), high (≥42). Cohen’s d = 2.53, *p* < 0.001.

**Table 6:** Retention of knowledge and practice improvements in the intervention group over time.

Time Point	Knowledge Scores	Practice Scores	*p*-Value (Knowledge)	*p*-Value (Practice)
Immediately post-intervention	20.62 ± 6.7	62.28 ± 4.1	<0.001	<0.001
1-Month Follow-Up	20.15 ± 6.9	61.95 ± 4.4	<0.001	<0.001
3-Month Follow-Up	19.72 ± 7.1	61.23 ± 4.7	<0.001	<0.001
6-Month Follow-Up	19.35 ± 7.2	60.76 ± 4.9	<0.001	<0.001

Values are presented as mean ± standard deviation. *p*-values are calculated by comparing each follow-up time point to the baseline scores using paired *t*-tests. Knowledge scores range from 0 to 48, and practice scores range from 0 to 73, with higher scores indicating better performance.

**Table 7:** Multivariate analysis of factors influencing post-intervention knowledge and practice scores.

**Variable**	**Knowledge Score**		**Practice Score**	
	**β (95% CI)**	***p*-Value**	**β (95% CI)**	***p*-Value**
Group (Intervention vs. Control)	12.46 (11.78, 13.14)	<0.001	22.22 (21.03, 23.41)	<0.001
Age (reference: <20)				
20–<30	0.05 (−0.62, 0.72)	0.883	0.08 (−1.11, 1.27)	0.895
30–<40	0.03 (−0.65, 0.71)	0.931	0.05 (−1.15, 1.25)	0.935
≥40	0.01 (−0.68, 0.70)	0.977	0.02 (−1.19, 1.23)	0.973
Years of Experience (reference: <5 years)				
5–<10 years	0.04 (−0.63, 0.71)	0.907	0.07 (−1.12, 1.26)	0.908
≥10 years	0.06 (−0.61, 0.73)	0.861	0.09 (−1.10, 1.28)	0.882
Educational Qualifications (reference: Technical Institute)				
Bachelor’s degree in nursing	0.21 (−0.47, 0.89)	0.545	0.35 (−0.84, 1.54)	0.563
Postgraduate Studies	0.25 (−0.43, 0.93)	0.470	0.40 (−0.79, 1.59)	0.509
Prior Training (Yes vs. No)	0.73 (0.03, 1.43)	0.041	1.28 (0.06, 2.50)	0.039
Baseline Score	0.42 (0.35, 0.49)	<0.001	0.38 (0.31, 0.45)	<0.001

β = regression coefficient; CI = confidence interval. Reference categories: age (<20 years), years of experience (<5 years), and educational qualifications (Technical Institute of Nursing). Baseline Score refers to the pre-intervention score for each respective outcome (knowledge or practice).

**Table 8:** Path analysis results—effects of PNE Model implementation on nurses’ knowledge and practice.

Pathway	Standardized Coefficient (β)	Standard Error (SE)	Critical Ratio (CR)	*p*-Value
Direct Effects				
PNE Model → Nurses’ Knowledge	0.70	0.05	14.00	<0.001
Nurses’ Knowledge → Practice	0.75	0.04	18.75	<0.001
PNE Model → Practice	0.25	0.06	4.17	<0.001
Indirect Effects				
PNE Model → Practice (via Knowledge)	0.53	0.04	13.25	<0.001
Total Effects				
PNE Model → Practice	0.78 (Direct + Indirect)			

## Data Availability

The data from this study are available upon request from the corresponding author due to privacy and ethical restrictions, as they involve sensitive patient information and professional performance data that cannot be shared publicly to protect confidentiality.
